# Correction: Application of soot discharged from the combustion of marine gas oil as an anode material for lithium ion batteries

**DOI:** 10.1039/d0ra90113j

**Published:** 2020-11-11

**Authors:** Hyun-Min Baek, Dae-Yeong Kim, Won-Ju Lee, Jun Kang

**Affiliations:** 2nd Fleet, ROK Navy Pyungtaek 17952 South Korea; Department of Mechanical Engineering, Tokyo Institute of Technology Meguro-ku Tokyo 152-8550 Japan kim.d.as@m.titech.ac.jp; Division of Marine Engineering, Korea Maritime and Ocean University 727 Taejong-ro, Yeongdo-gu Busan 49112 Republic of Korea junkang@kmou.ac.kr skywonju@kmou.ac.kr

## Abstract

Correction for ‘Application of soot discharged from the combustion of marine gas oil as an anode material for lithium ion batteries’ by Hyun-Min Baek *et al.*, *RSC Adv.*, 2020, **10**, 36478–36484.

The authors regret that a funder was omitted that should have been acknowledged. This acknowledgement is as follows:

This research was supported by the Ministry of Education of Republic of Korea and the National Research Foundation of Korea (NRF-2019R1G1A1005342).

The Royal Society of Chemistry apologises for these errors and any consequent inconvenience to authors and readers.

## Supplementary Material

